# BUFFERING EFFECT OF MARRIAGE ON DAILY WORK STRESS–INFLAMMATION LINK: LONGITUDINAL ANALYSIS OF THE MIDUS STUDY

**DOI:** 10.1093/geroni/igae098.2868

**Published:** 2024-12-31

**Authors:** Jeongmin Park, David Almeida, Alexis Santos-Lozada

**Affiliations:** The Pennsylvania State University, University Park, Pennsylvania, United States; Pennsylvania State University, University Park, Pennsylvania, United States; The Pennsylvania State University, University Park, Pennsylvania, United States

## Abstract

**Aims:**

This study investigates how frequency of daily work stress predicts inflammation, measured by C-reactive protein levels (CRP), in employed middle-aged and older adults. It explores Relationship Buffering Hypothesis by assessing how marital status and quality of marital relationships moderate this stress-inflammation link. Additionally, it examines role of age and gender. Based on Socio-Emotional Selectivity Theory, we hypothesize that relationship buffering will be greater for older workers.

**Methods:**

Data were from Midlife in the United States (MIDUS) waves 2 and 3, samples include 314 employed individuals over 40 (at wave 3), participating in both National Study of Daily Experiences and Biomarker projects. CRP was measured during laboratory visits, whereas daily work stress was computed as the mean of 8-day diary responses of binary stress events at workplace ranging from 0 (not work stressors) to 1 (work stressors every day).

**Results:**

Linear regression revealed that frequent daily work stress predicts higher CRP a decade later, controlling for initial CRP, age, gender, education, and race (B = 4.49, p < 0.01). Marital status (B = -8.35, p < 0.01) and relationship quality among married individuals (B = -1.41, p < 0.05) moderated this relationship. No significant three-way interactions with age or gender were found. Conclusions: The daily work stress-inflammation link varied by status and relationship quality of marriage. Specifically, non-married individuals and those experiencing lower relationship quality within marriage showed a heightened stress-inflammation link, underscoring the protective role of strong marital relationships against stress-induced inflammation, in alignment with the Relationship Buffering Hypothesis.

